# Leukocytes Are Recruited through the Bronchial Circulation to the Lung in a Spontaneously Hypertensive Rat Model of COPD

**DOI:** 10.1371/journal.pone.0033304

**Published:** 2012-03-21

**Authors:** Benjamin B. Davis, Yi-Hsin Shen, Daniel J. Tancredi, Vanessa Flores, Ryan P. Davis, Kent E. Pinkerton

**Affiliations:** Center for Health and the Environment, University of California Davis, Davis, California, United States of America; Comprehensive Pneumology Center, Germany

## Abstract

Chronic obstructive pulmonary disease (COPD) kills approximately 2.8 million people each year, and more than 80% of COPD cases can be attributed to smoking. Leukocytes recruited to the lung contribute to COPD pathology by releasing reactive oxygen metabolites and proteolytic enzymes. In this work, we investigated where leukocytes enter the lung in the early stages of COPD in order to better understand their effect as a contributor to the development of COPD. We simultaneously evaluated the parenchyma and airways for neutrophil accumulation, as well as increases in the adhesion molecules and chemokines that cause leukocyte recruitment in the early stages of tobacco smoke induced lung disease. We found neutrophil accumulation and increased expression of adhesion molecules and chemokines in the bronchial blood vessels that correlated with the accumulation of leukocytes recovered from the lung. The expression of adhesion molecules and chemokines in other vascular beds did not correlate with leukocytes recovered in bronchoalveolar lavage fluid (BALF). These data strongly suggest leukocytes are recruited in large measure through the bronchial circulation in response to tobacco smoke. Our findings have important implications for understanding the etiology of COPD and suggest that pharmaceuticals designed to reduce leukocyte recruitment through the bronchial circulation may be a potential therapy to treat COPD.

## Introduction

Chronic obstructive pulmonary disease (COPD) is the fourth leading cause of death in the United States [1], and 80–90% of COPD cases can be attributed to smoking [2]. COPD is characterized by airflow limitation presented as either chronic bronchitis, emphysema or both. Chronic bronchitis is distinguished by excessive mucous production, airway wall thickening, epithelial squamous metaplasia, and leukocyte recruitment to airway walls [3]. Emphysema is characterized by airspace enlargement and parenchymal destruction [4].

Leukocytes recruited to the lung in response to tobacco smoke contribute to the development of both airway and alveolar manifestations of COPD by releasing reactive oxygen metabolites and proteolytic enzymes. Positive feedback loops are triggered that perpetuate leukocyte recruitment, subsequent airway epithelial damage and airspace enlargement after smoking cessation [5]. Leukocytes are recruited to inflamed tissues via adhesion molecule and chemokine expression when an acute inflammatory stimulus triggers increased adhesion molecule and chemokine expression by vascular endothelial cells and adjacent tissue. Adhesion molecules act by capturing leukocytes from the blood stream. Chemokines facilitate transmigration of leukocytes out of the blood vessels and to the inflamed tissue [6].

The location of leukocyte emigration into the lung in response to tobacco-smoke is unknown. However, some evidence from smoke-induced COPD suggests that the bronchial blood vessels may play a role in leukocyte recruitment. Bronchial biopsies from COPD patients have demonstrated increased E-selectin, ICAM, IL-8 and MCP-1 in the bronchial blood vessels or submucosa [7]–[10].

In this work, we used a spontaneously hypertensive (SH) rat model of COPD to investigate the mechanism and location of smoke-induced leukocyte recruitment to the lung.

## Materials and Methods

### Animals

Twelve-week-old male SH rats were purchased from Charles River Laboratories (Portage, MI). Upon arrival, all animals were housed in polycarbonate cages under a 12-hour light-dark cycle with continuous access to food and water. Animals were acclimated to the new housing environment for one week before tobacco smoke exposure began. All animals were handled according to the U.S. Animal Welfare Acts, and all procedures were performed under the supervision of the University Animal Care and Use Committee (University of California, Davis, protocol number 15956). Two animals from the 4 week smoke exposure group and one rat from the 12 week smoke exposure group were removed from the study due to lethargy and significant weight loss.

### Tobacco Smoke Exposure

Groups of 6 SH rats each were exposed to filtered air or to tobacco smoke at a concentration of approximately 80–90 mg/m^3^ total suspended particulates (TSP) for 6 hours/day, 3 days/week, for either 3 days, 4 weeks, or 12 weeks. A total of four experiments were performed: two three-day exposures, one four-week exposure and one twelve-week exposure. To increase the power and make this study possible we utilize two three day smoke exposure studies. The two three-day exposure experiments were designed as a 2×2 factorial to evaluate two binary factors, tobacco smoke (TS) vs. filtered air (FA) and soluble epoxide hydrolase inhibitor (sEHI) vs. no drug, with 6 animals per cell. Treatment with the drug had no effect on leukocyte recruitment or adhesion molecule expression [11]. Whole body exposure to cigarette smoke was done using a TE10 smoke exposure system [12] that combusts 3R4F research cigarettes (Tobacco and Health Research Institute, University of Kentucky, KY) with a 35 ml puff volume of 2 seconds duration, once each minute following the Federal Trade Commission smoking standard. In addition to TSP, exposure conditions were monitored daily for both nicotine and carbon monoxide concentrations.

### Tissue Preparation

SH rats were anesthetized with an overdose of sodium pentobarbital 18–20 hours following the final day of exposure with the exception of one of the 3-day studies in which the rats were sacrificed 1–3 hours post-exposure. The trachea was cannulated, the left lung bronchus tied, and the right lung lavaged with Ca^2+^/Mg^2+^-free phosphate buffered saline (PBS) (pH 7.4) or Hank's buffered salt solution (HBSS) with a volume calculated from the body weight, according to the equation of 35 ml/kg body weight ×2/3 of filtered air control rats. Bronchoalveolar lavage (BAL) was performed using a three-in/three-out pattern of intratracheal instillation and removal with the same PBS aliquot in order to enrich total cell and protein recovery. BAL fluid (BALF) was collected in tubes and kept on ice prior to processing. The lavaged lung lobes were frozen in liquid nitrogen and stored at −80°C until use. For histology, the suture on the left lung bronchus was released and the lung was inflated with 4% paraformaldehyde at 30 cm water pressure for 1 hour, followed by storage of the inflation-fixed lung immersed in fixative.

### BALF Analysis

The BALF was centrifuged at 250×g for 10 minutes at 4°C to separate cells from the supernatant fluid (16). After centrifugation, the cell pellet was resuspended in Ca^2+^/Mg^2+^-free PBS or HBSS. The cell suspension was assayed for cell viability as determined by trypan blue exclusion. Total cell number was determined using a hemocytometer. Cytospin slides (Shandon, Pittsburgh, PA) were prepared using aliquots of cell suspension that were then stained with Hema 3 (Fisher Scientific, Pittsburgh, PA). Cell differentials in BALF were assessed by counting macrophages, neutrophils, lymphocytes, and eosinophils on cytocentrifuge slides using light microscopy (over 500 cells counted per sample). The proportion of each cell type was multiplied by the total cell number per ml to determine total neutrophils, macrophages, lymphocytes and eosinophils per ml.

### Immunohistochemistry

Immunohistochemistry was performed using transverse lung tissue slices containing the first and second intrapulmonary airway generations. Five micron thick sections were cut from paraffin-embedded tissue blocks on a microtome. Sections were placed on glass slides and baked overnight at 37°C. Sections were subsequently deparaffinized in toluene and hydrated through a graded series of alcohol. Antigen retrieval consisted of heat treatment by decloaker (123°C for 2 min and 83°C for 10 sec, Decloaking Chamber, Biocare Medical, Concord, CA) with sections in EDTA (pH 8, IHC Select). Incubation with primary antibodies was for one hour for all sections. Goat anti-rat ICAM (0.01 µg/ml), VCAM (0.1 µg/ml), and E-selectin (0.1 µg/ml) antibodies (R&D Systems, Minneapolis, MN) and rabbit anti-rat MCP-I (0.1 µg/ml) and MIP-2 (0.01 µg/ml) antibodies (Abcam, Cambridge, MA) were used as primary antibodies. The following was used for antibody visualization: 1) biotinylated anti-Goat IgG (Vector BA5000, Burlingame, CA) Liquid DAB+Substrate Chromogen System (Dako K3468, Carpinteria, CA) for rabbit anti-rat antibodies, 2) biotinylated anti-Goat IgG (Vector BA5000) with HRP Streptavidin (Zymed 50-209Z, South San Francisco, CA) for goat anti-rat antibodies, and 3) Liquid DAB+Substrate (Dako K3468) for both antibody types. Sections were then hematoxylin counterstained, dehydrated in ethanols and mounted with Clear Mount (American Tech Master Scientific, Inc., Lodi, CA). For hematoxylin and eosin staining, sections were stained with the following American Master Tech Scientific materials: Harris Hematoxylin, Differentiating Solution, Bluing Solution, and Eosin Y Stain.

For immunohistochemical data analysis, the entire lung tissue section composed of airways, blood vessels, parenchyma and pleura was evaluated for each animal by a blinded observer for adhesion molecule or chemokine staining with two different concentrations of antibody. Staining intensities that had unambiguous cellular labeling at either antibody concentration were evaluated at the airway (proximal and distal), pulmonary artery and vein, lung parenchyma and pleura. In locations where staining differences could be determined, a score of zero was given to the rat with the least staining and a score of five was given to the rat with the most prominent staining based on both the frequency and intensity of staining at each anatomical location. In a 40% subsample that was also scored by a second blinded observer, inter-rater reliability was estimated to be 94%. This technique made it possible to analyze the complete tissue section for changes in expression in multiple locations simultaneously.

### Bronchial blood vessel neovasculazation

Bronchial blood vessels were counted in hematoxylin and eosin stained slides and reported as blood vessels per mm airway. The circumference of airways was measured with Image J software.

### Mean linear intercept

Non-overlapping fields of hematoxylin and eosin stained sections consisting of only alveolar tissues (i.e., alveolar ducts and alveoli) were captured. The volume fraction (V_v_) of alveoli and alveolar ducts were determined by point counting using the formula: V_v_ = P_p_ = P_n_/P_t_ where P_p_ is the point fraction of P_n_, the number of test points hitting the structure of interest, divided by P_t_, the total points hitting the reference space (parenchyma). In addition, the mean linear intercept (MLI) length of the alveolar airspace was determined by counting the number of intercepts a random test line of known length made with alveolar septa. MLI is expressed as the average length of the line between intercepts.

### Statistical analysis

Duration-specific (3 day, 4-week and 12-week) contrasts in mean levels of study outcomes between tobacco-smoke and filter-air exposed rats were assessed and compared by fitting ANOVA or regression models that included main effects for smoking and duration and interaction terms for duration and smoking. To minimize confounding due to the additional complexity of the two three-day experiments, a variety of approaches were used. For the mean linear intercept outcome, the analysis was restricted to just the non-drug exposed animals from studies with the 18–20 hour post-exposure sacrifice interval. For comparing mean levels of the other outcomes, ANOVA/regression models included independent binary indicator variables for the drug factor (coded ‘1’ if exposed to sEHI and ‘0’ otherwise) and for experiment factor (coded ‘1’ for the 3-day experiment with the shorter post-exposure sacrifice interval and ‘0’ otherwise), to statistically adjust for variation due to these two nuisance factors.

To estimate and compare the role of different vascular bed locations on leukocyte recruitment, we correlated location-specific smoke-induced adhesion molecule and chemokine expression levels with numbers of leukocytes recovered in the BALF. Animals from the smoke-exposed groups in both experiments with three day exposure durations were analyzed together to increase the power of this analysis. To statistically adjust for extraneous variation due to between-experiment effects and to the drug factor in the 3-day experiments, adhesion molecule and chemokine expression scores were rank-transformed within blocks defined by unique combinations of experiment and drug exposure for these correlation analyses [13]. The Spearman correlation of the smoke-induced adjusted ranks of adhesion molecule or chemokine expression at each location with adjusted ranks of leukocytes and neutrophils recovered in the BALF is reported.

To assess and compare the effects of smoke exposures of various durations on blood cell counts and on adhesion molecule and chemokine expression levels in or around the bronchial blood vessels, mean levels of outcomes were compared using analysis of variance methods [14]. Blood cell counts were log-transformed to reduce skewness and analyzed using ANOVA models as described above. For each blood cell count outcome, separate models were specified for the 3-day exposure experiments and for the remainder of the data. To account for the non-normality of each expression level outcome, the data from all of the experiments were pooled, rank-transformed and then analysed in a single model, with the statistical significance of the duration specific smoking vs. filtered contrasts (and between-duration comparisons of these contrasts) assessed using permutation tests in order to ensure the robustness and validity of our inferences [15]. A Monte Carlo approximation (based on 13,000 replications) to the permutation distribution was generated by randomly permuting the smoking factor within blocks defined by the combination of study and drug exposure using the standard re-sampling procedures available in Stata (StataCorp LP, College Station, Texas). Sensitivity analyses were also conducted to verify that conclusions were not substantively affected by methods used to account for the more complex 3-day experimental data.

## Results

### Cigarette smoke induces extensive damage to the bronchial epithelium

**Figure 1:**
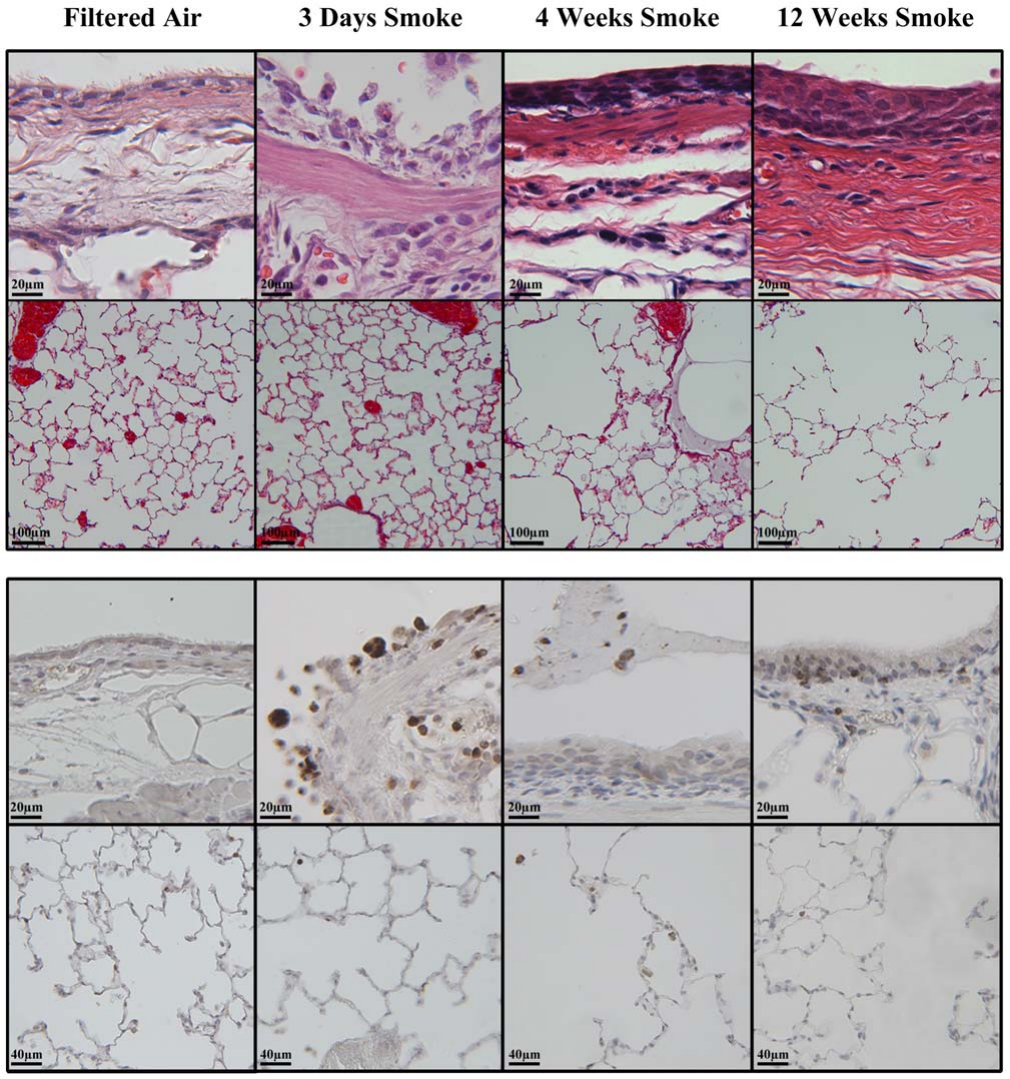
Tobacco smoke induced damage to the lung. Cigarette smoke-induced damage was evaluated in hematoxylin stained lung tissue sections (top). Cigarette smoke-induced neutrophil infiltration was evaluated using myeloperoxidase staining of lung tissue sections to confirm the location of neutrophils in the vasculature and lung tissues (bottom). Tissue sections are from SH rats exposed to 6 hours of tobacco smoke per day for 3 days, 4 weeks or 12 weeks.

**Figure 2:**
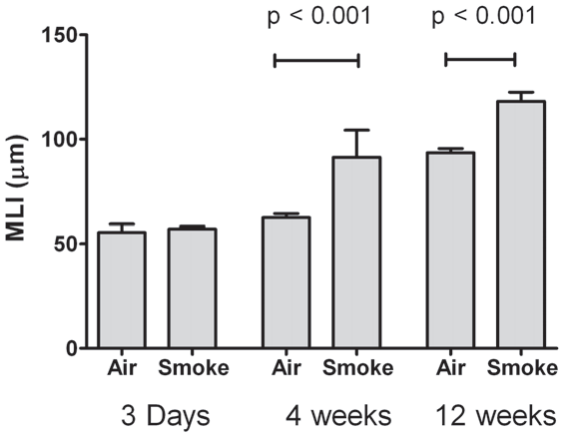
Mean linear intercept. Mean linear intercept is reported as a measurement of alveolar airspace enlargement within the lung parenchyma after 3 days, 4 weeks or 12 weeks of filtered air or tobacco smoke exposure.

Exposure of rats to smoke for 3 days caused extensive damage to the bronchial airway epithelium that was associated with epithelial cell death and sloughing as well as neutrophil infiltration of the bronchial wall and epithelium in the absence of notable edema (Figure 1). There was no obvious cell death and no increase in neutrophil infiltration within the alveolar parenchyma. After 4 weeks of tobacco smoke exposure, there were extensive areas of squamous epithelial metaplasia along the central bronchial airway. In addition, more distal airways demonstrated an abundance of goblet cells lining the luminal surface and increased cellularity within the airway wall, including the presence of neutrophils and accumulation of macrophages and neutrophils within the lumen of the airways frequently enmeshed in a thick mucous lining. Some mucous secretions were also observed to extend down into the alveolar airspace; however, this may be due to fixative pushing mucous from the airway to the parenchyma. After 12 weeks of smoke exposure, there were large areas of keratinizing, stratified squamous epithelium and infiltration of neutrophils in the bronchial airways. Mucous secretions continued to be found to extend into the alveolar airspace, and occasional alveolar ducts showed marked distension. Airspace enlargement was measured by mean linear intercept. Consistent with the pathology observed in the hematoxylin and eosin staining, there was no increase in airspace enlargement after 3 days of smoke exposure compared to filtered air (3-day TS vs. FA contrast in mean MLI = 1.7; 95% CI = −9.8 to 13.3; p = 0.77). However, there were significant increases in airspace enlargement after 4 and 12 weeks of tobacco smoke exposure (Figure 2) compared to control (4-week contrast = 28.9; 95% CI = 16.6 to 41.2; p<0.001; 12-week contrast = 24.7; 95% CI = 13.2 to 36.2; p<0.001). Tobacco-smoke versus filtered air contrasts are statistically significantly different between durations of exposure (p<0.01 for F-test of duration X smoke interaction). The mean linear intercept was increased in the 12 week experiment as compared to the 4 week and 3 day experiments independent of smoke exposure. For example, among filtered air exposed animals, the difference in mean linear intercept between 12-week versus 4-week exposed animals is 31.1 (95% CI = 20.1 to 42.1; p<0.001).

### Leukocytes are recruited to the lung following acute tobacco smoke exposure

To determine which blood vessels facilitate leukocyte emigration from the blood into the lung, we first confirmed that acute exposure to tobacco smoke increased leukocyte recruitment to the lung. Following three days of smoke exposure geometric mean (95% CI) concentrations (cell count per ml) of total leukocytes and neutrophils recovered from bronchoalveolar lavage fluid (BALF) were 11.9×10^4^ (8.3×10^4^ to 17.2×10^4^) and 5.3×10^4^ (3.1×10^4^ to 9.2×10^4^), respectively, and were significantly increased compared to the geometric mean (95% CI) concentrations observed in filtered air exposed rats 3.2×10^4^ (2.7×10^4^ to 3.9×10^4^) leukocytes per ml and 0.15×10^4^ (0.11×10^4^ to 0.19×10^4^) neutrophils per ml.

### Increased adhesion molecule and chemokine expression in the bronchial blood vessels following acute tobacco smoke exposure

**Figure 3:**
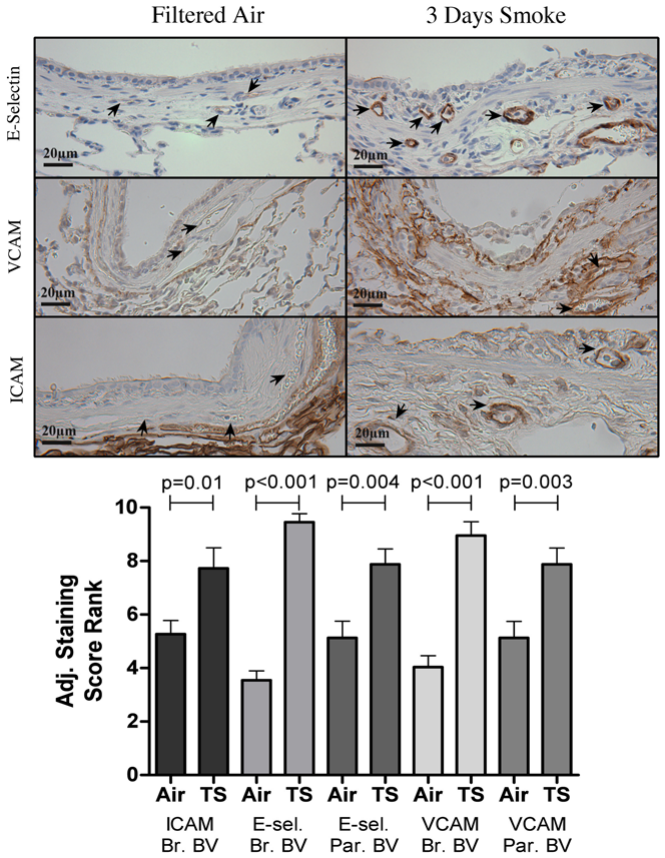
Tobacco smoke induced adhesion molecule expression. Top) Representative pictures of immunohistochemical (IHC) staining of E-selectin, VCAM, and ICAM in the bronchial wall of SH rats exposed to filtered air or 3 days of tobacco smoke. Arrows indicate bronchial blood vessels. Bottom) IHC staining intensity of the adhesion molecules E-selectin, VCAM, and ICAM after 3 days of tobacco smoke as scored by blinded ranking. Br BV: bronchial blood vessel; Par BV: parenchymal blood vessel. Brackets indicate comparisons between filtered air exposure and tobacco smoke exposure. (Note: Adhesion molecules and chemokine staining scores were reranked putting them on a 1–12 scale. See methods).

**Figure 4:**
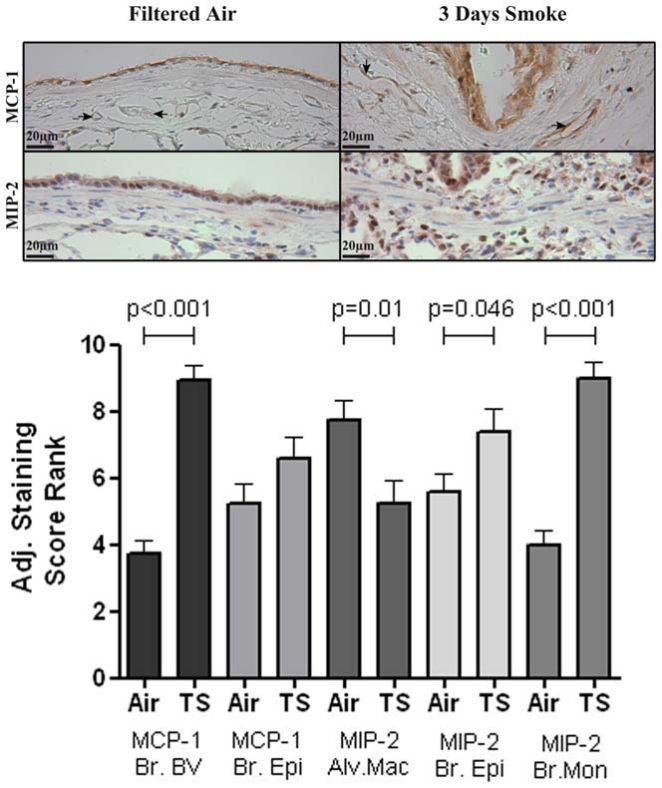
Tobacco smoke induced chemokine expression. Top) Representative pictures of immunohistochemical (IHC) staining of MCP-1 and MIP-2 in the bronchial wall of SH rats exposed to filtered air or 3 days of tobacco smoke. Bottom) IHC staining intensity of the chemokines MCP-1 and MIP-2 after 3 days of tobacco smoke as scored by blinded ranking. Br BV: bronchial blood vessel; Br Epi: bronchial epithelial cells; Alv. Mac: alveolar macrophages; Br Mac: macrophages in the bronchial wall. Brackets indicate comparisons between filtered air exposure and tobacco smoke exposure. (Note: Adhesion molecules and chemokine staining scores were reranked putting them on a 1–12 scale. See methods).

Using immunohistochemistry, we analyzed the lung for increased expression of adhesion molecules and chemokines that facilitate leukocyte emigration following 3 days of tobacco smoke exposure. Expression of the adhesion molecules E-selectin, VCAM, and ICAM and the chemokines MCP-1 and MIP-2 were evaluated and found expressed in site-specific regions of the lung (Figures 3 and 4). E-selectin and VCAM expression was significantly increased in the bronchial blood vessels and pulmonary blood vessels of the parenchyma following tobacco smoke exposure (Figure 3). ICAM was also found significantly increased in the bronchial blood vessels (Figure 3) but changes in the parenchyma blood vessels were not detected (data not shown). Tobacco smoke-induced increases in the expression of MCP-1 and MIP-2 were detected in the vicinity of the bronchial wall. MCP-1 was significantly increased in bronchial blood vessels, and MIP-2 was significantly increased in the epithelium and macrophages of the bronchial wall (Figure 4). On the other hand, alveolar macrophages had decreased MIP-2 expression following tobacco smoke exposure. Somewhat surprisingly, there was no detection of smoke-induced expression of the adhesion molecules or chemokines in the alveolar capillaries, and no detectable increases in the expression of the adhesion molecules or chemokines were observed in the pulmonary artery, large pulmonary vein adjacent to the bronchus, or pleura (results not shown).

To determine if the smoke induced increases in adhesion molecule and chemokine expression at a location are responsible for leukocyte recruitment to the lung we correlated expression scores after 3-days of smoke exposure with the number of leukocytes recovered in the BALF. E-selectin, ICAM and MCP-1 in the bronchial blood vessels and MIP-2 expression in mononuclear cells within the bronchial wall significantly correlated with total leukocytes (Table 1) and neutrophils recovered from BALF (Table 2). The majority of the leukocytes recruited to the lung in response to 3 days of tobacco smoke were neutrophils. The correlation of the bronchial expression of E-selectin, ICAM, and MIP-2, which are known to cause leukocyte recruitment, strongly suggests that the majority of leukocytes are recruited from the bronchial blood vessels during the early stages of this tobacco smoke induced model of COPD.

**Table 1:** Adhesion molecule and chemokine expression at specific locations correlate with leukocytes recovered from the BALF.

Adhesion molecule		correlation coef	
	location	rho (95% CI)	p value
ICAM	Bronch. BV	0.55 (0.31–0.80)	<0.001*
E-Selectin	Bronch. BV	0.54 (0.21–0.86)	0.001*
E-Selectin	Paren. BV	0.10 (−0.26–0.47)	0.6
VCAM	Bronch. BV	0.05 (−0.35–0.45)	0.8
VCAM	Paren. BV	0.15 (−0.26–0.57)	0.5
MCP-1	Bronch. BV	0.36 (0.03–0.69)	0.03*
MCP-1	Bronch. EC	0.18 (−0.18–0.56)	0.3
MIP-2	Alveolar Mac	−0.34 (−0.69–0.004)	0.053
MIP-2	Bronch. EC	−0.04 (−0.44–0.36)	0.9
MIP-2	Bronch. Mon	0.41 (0.12–0.70)	0.006*

**Table 2:** Neutrophil specific adhesion molecule and chemokine expression at specific locations correlate with neutrophils recovered from the BALF.

Adhesion molecule		correlation coef	
	location	rho (95% CI)	p value
ICAM	Bronch. BV	0.57 (0.35–0.80)	<0.001*
E-Selectin	Bronch. BV	0.45 (0.10–0.80)	0.012*
E-Selectin	Paren. BV	0.31 (−0.05–0.67)	0.09
MIP-2	Alveolar Mac	−0.34 (−0.68–0.01)	0.054
MIP-2	Bronch. EC	0.10 (−0.27–0.46)	0.593
MIP-2	Bronch. Mon	0.63 (0.43–0.84)	<0.001*

### Tobacco smoke increases the vascularization of the bronchial wall

**Figure 5:**
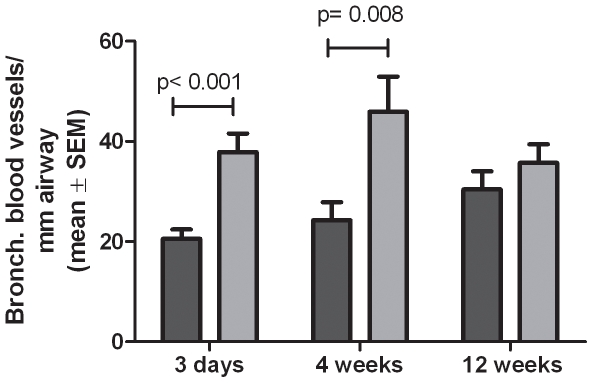
Smoke increases neovasculization. Bronchial blood vessels per mm airway lumen at 3 days, 4 weeks and 12 weeks smoke exposure.

Bronchial blood vessels in hematoxylin and eosin stained sections were quantified. Compared to filtered air controls, tobacco smoke exposure significantly increases the concentration (#/mm airway) of bronchial blood vessels in the bronchial walls after 3 days of exposure and after 4 weeks exposure, but not after 12 weeks exposure (Figure 5). Between-duration comparisons of the duration-specific contrasts were not statistically significant, however.

### Increases in leukocyte recruitment, adhesion molecule expression and chemokine expression diminish with long term smoke exposure

**Figure 6:**
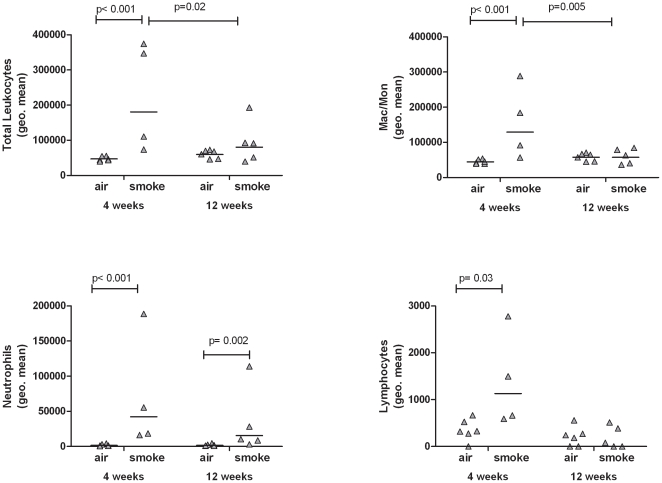
Leukocytes recovered from the BALF of SH rats after 3 days, 4 weeks, or 12 weeks of tobacco smoke exposure. Total leukocytes, monocyte/macrophages neutrophils, and lymphocytesrecovered from the BALF after 3 days, 4 weeks, or 12 weeks of tobacco smoke exposure.

Total leukocytes, neutrophils, and macrophages were measured in BALF following 3 days, 4 weeks and 12 weeks of tobacco smoke exposure. Compared to filtered air exposure of similar duration, 3 days (see previous result sections) and 4 weeks of tobacco smoke exposure resulted in significantly increased total leukocytes recovered from BALF (Figure 6). However, there was no significant difference in total BALF leukocytes between animals exposed to filtered air and tobacco smoke after 12 weeks of exposure. The effect of tobacco smoke vs filtered air was statistically significantly different at 12 weeks versus 4-weeks (F-ratio for interaction = 6.28; p = 0.02). Compared to rats exposed to filtered air, rats exposed to tobacco smoke had significantly more neutrophils in BALF at all time points. The magnitudes of the tobacco smoke effect were not statistically different at 4-weeks compared to 12-weeks, however.. Macrophages recovered from BALF were similar to total leukocytes, being significantly increased after 3 days and at 4 weeks of tobacco smoke exposure but not after 12 weeks (and with the 4-week TS vs. FA contrast significantly different from the 12-week contrast, by the F-test). Lymphocytes made up less than 2% of the total cells and were increased by tobacco smoke at 4-weeks, but not at 12-weeks. (Figure 6).

**Figure 7:**
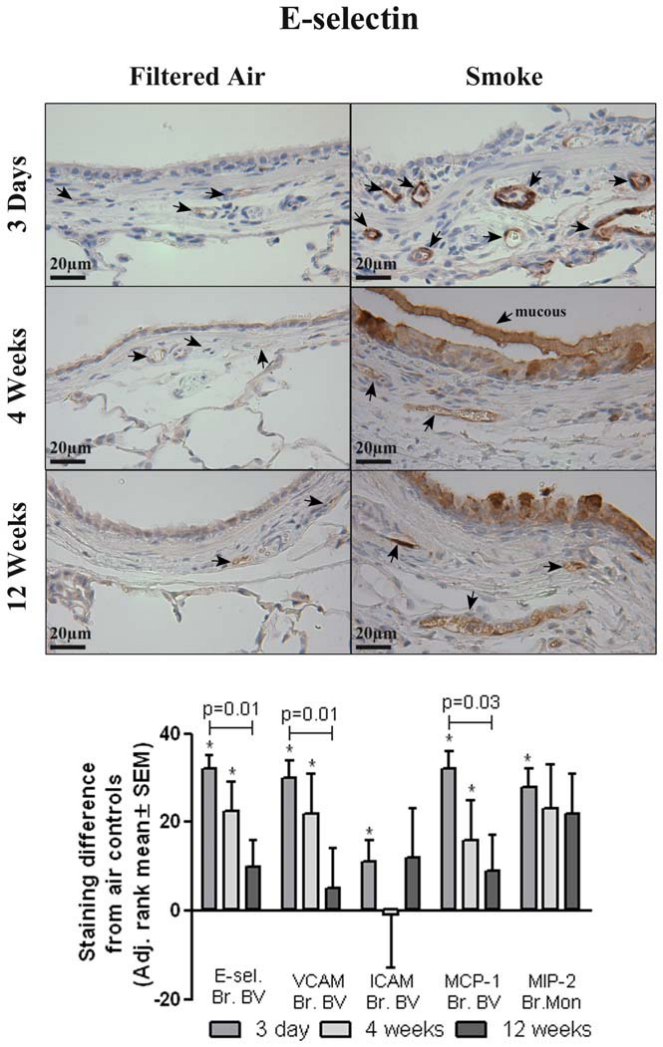
Adhesion molecule and chemokine expression in and around the bronchial blood vessels following 3 days, 4 weeks, or 12 weeks of tobacco smoke exposure. Top) Representative pictures of immunohistochemical staining of E-selectin in the bronchial wall of SH rats exposed to filtered air or tobacco smoke for 3 days, 4 weeks, or 12 weeks. Arrows indicate bronchial blood vessels. Bottom) IHC staining intensity of E-selectin, VCAM, ICAM, MCP-1, and MIP-2 after 3 days, 4 weeks, or 12 weeks of tobacco smoke as scored by blinded ranking. The difference from control staining is reported as mean ± SEM, where the mean control staining at each location is set to zero. * *p* value<0.05 vs. filtered air. Brackets indicate comparisons between 3 days, 4 weeks and 12 weeks of smoke exposure. (staining scores were reranked prior to analysis changing the scale, see methods).

We also evaluated adhesion molecule and chemokine expression in the bronchial blood vessels after 3 days, 4 weeks and 12 weeks of tobacco smoke exposure. Tobacco-smoke versus filtered air contrasts in E-selectin, VCAM and MCP-1 expression levels in the bronchial blood vessels were statistically significant at 3 days and at 4 weeks, but not at 12 weeks. The comparison of the 3-day and the 12-week contrasts was statistically significant for each of these outcomes. Immunohistochemical staining of E-selectin at 3 days, 4 weeks and 12 weeks is shown in Figure 7 (top), with the staining scores of the other adhesion molecules and chemokines in or around the bronchial blood vessels shown at the bottom of Figure 7.

## Discussion

We find that acute tobacco smoke exposure causes neutrophil accumulation and increased expression of adhesion molecules and chemokines in the bronchial blood vessels. This increased expression of adhesion molecules and chemokines correlated with the accumulation of leukocytes recovered from the lung. The expression of adhesion molecules and chemokines in other vascular beds did not correlate with leukocytes recovered in BALF. These findings suggest that leukocytes are recruited primarily through the bronchial circulation in response to tobacco smoke in a rat model of COPD.

The respiratory system is unique compared to other organ systems because it utilizes both classical and alternative mechanisms to recruit leukocytes [16]–[19]. In the classic model of leukocyte recruitment, an acute inflammatory stimulus triggers increased adhesion molecule and chemokine expression by vascular endothelial cells and adjacent tissue that facilitates leukocyte adhesion and subsequent migration into the inflamed tissue. Selectin family adhesion molecules expressed on activated vascular endothelial cells assist with the initial capture of leukocytes from the blood stream by tethering, after which leukocytes roll along the vascular endothelium via transient bonds with selectins and IgG family adhesion molecules, including ICAM and VCAM. As the leukocytes roll, chemokines, such as MCP-1 and IL-8 (MIP-2 in rats), released by the vascular endothelial cells and surrounding tissues activate the leukocytes, resulting in their increased binding affinity to ICAM and/or VCAM. The increased leukocyte binding affinity for ICAM and/or VCAM facilitates the arrest and eventual transmigration of leukocytes through blood vessels. Leukocytes continue to migrate, following a chemokine concentration gradient to regions of inflamed lung tissue (reviewed by Malik et al. [6]).

The alternative mechanism of leukocyte recruitment occurs in the alveolar capillaries via a unique mechanism that is independent of selectins and ICAM. During an acute inflammatory event, the majority of pulmonary leukocytes are in alveolar capillaries. This is likely due to the small size of the alveolar capillaries slowing the passage and increasing the concentration of neutrophils to 35–100 times greater in alveolar capillaries than in the systemic circulation. The narrow passages of the alveolar capillaries eliminate the need for neutrophil capture by adhesion molecules. Several studies using bacteria or bacterial products or acid instillation have demonstrated ICAM and/or E-selectin-independent transmigration of leukocytes [20]–[25], suggesting that in general, leukocytes extravasate from the alveolar capillaries during an inflammatory response.

Results from this study indicate that leukocytes are being classically recruited through the bronchial blood vessels in response to tobacco smoke exposure because the time points with the greatest increase in BALF leukocytes are the time points with the greatest adhesion molecule/chemokine expression in the bronchial blood vessels. After a 12-week smoke exposure, rats had significant increases in total leukocytes in BALF, but the increase was much less robust as compared to after a 3-day smoke exposure. Furthermore, adhesion molecule and chemokine expression in or around the bronchial blood vessels of the 12 week exposure group was generally increased, but to a lesser extent than following the 3 day exposure group. This further supports the concept that bronchial blood vessels are a major contributor to leukocyte extravasation in response to tobacco smoke during the acute or initial genesis of the COPD process as well as during all stages of COPD progression. These findings suggest that unlike other lung inflammatory stimuli, leukocytes are recruited primarily through the bronchial circulation in response to tobacco smoke. Several studies have demonstrated alveolar capillary recruitment in response to systemic inflammation or intralobular instillation that delivers inflammatory stimuli closer or deeper to the alveolar region [20]–[25]. We find that tobacco smoke causes initial damage in the bronchial epithelium with the greatest changes noted in the proximal or central airways. We have further noted leukocytes appear to be emigrating from those blood vessels in the bronchial walls of these central airways (Figure 1). It is not surprising that tobacco smoke extensively injures the bronchial epithelium, since it is the first tissue that the smoke encounters in the lower respiratory tract. Likewise, since injury triggering the release of inflammatory mediators leads to increased expression of adhesion molecules and chemokines [26] responsible for leukocyte recruitment to sites of inflammation, we would expect leukocyte recruitment to occur through blood vessels nearest to these sites of damage.

Recently, we reported damage to airway epithelial cells in SH rats repeatedly exposed to tobacco smoke for three months. Damage was evidenced by apoptosis and neutrophil infiltration in the bronchial wall with little apoptosis detected in the parenchyma [27]. Moreover, repeated long-term smoke exposure causes SH rats to develop epithelial squamous metaplasia, airway wall thickening and airspace enlargement [28]. We now demonstrate that these same rats have significant smoke-induced increases in neutrophils present in the lungs compared to filtered air control rats.

It is possible that long-term smoke exposure may result in the recruitment of neutrophils and other leukocytes through the alveolar capillaries in the absence of enhanced adhesion molecules and chemokines measured in this study. After 4 and 12 weeks of tobacco smoke exposure, we observed signs of alveolar damage as well as the presence of neutrophils within the lung parenchyma. However, the majority of neutrophils observed for each time point studied remained within the bronchial wall. VCAM and MIP-2 expression in the bronchial wall continued to be significantly increased relative to controls, indicating that the bronchial circulation remained a likely site of leukocyte emigration. Future work is needed to determine the relative contribution, if any, of leukocyte recruitment to the lung via the alveolar capillaries during long-term smoke exposure.

Additionally, we found exposure to tobacco smoke for 3 days and 4 weeks increased the number of bronchial blood vessels. It is possible that increased vascularization further enhances recruitment of leukocytes through the bronchial blood vessels. However, unlike adhesion molecule and chemokine expression, a causal relationship between neovasculazation and leukocyte recruitment has not been established. It is also possible that more bronchial blood vessels are needed because of increased metabolic requirements of the inflamed tissue.

This study further establishes the validity of repeated smoke exposure in the SH rat as a viable model of chronic obstructive lung disease by demonstrating similarities in the molecular mechanisms of leukocyte recruitment observed in human COPD. Repeated, intermittent exposure to tobacco smoke in SH rats can serve as an excellent animal model of human chronic lung inflammatory disease due to the fact SH rats possess many of the characteristics found in human COPD, including sustained recruitment of leukocytes to the lung with neutrophil infiltration of the bronchial wall, bronchial epithelial cell apoptosis, airway wall thickening, epithelial cell squamous metaplasia, goblet cell hypertrophy and mucous hypersecretion. Increases in inflammatory cytokines, oxidative stress, and protease activity have also been demonstrated, as well as airspace enlargement [28], [29]. Furthermore, in the work presented here, we demonstrate that E-selectin, ICAM, MCP-1, and IL-8 homologue MIP-2 expression are increased in the bronchial wall similar to that observed in bronchial biopsies from patients with COPD [7]–[9], [30].

These inflammatory and histopathological patterns noted in the SH rat over time further demonstrate the utility of this model for screening possible pharmaceuticals to treat COPD. Unlike other rodent models found in the literature, we see chronic inflammatory changes to the lung in as little as four weeks. It is now possible to determine whether a drug is reducing leukocyte recruitment to the lung by observing the effects of the drug on the adhesion molecules and chemokines in or around the bronchial blood vessels.

While the SH model is able to produce inflammatory changes to the lung that are similar to COPD in a short period of time, it differs from COPD in a couple of ways. The SH rat model has a very strong initial inflammatory cell recruitment that involves mostly neutrophils and monocytes. After 12 weeks of smoke exposure by SH rats, the ratio of inflammatory cells is still skewed toward a neutrophilic response with a less pronounced adaptive response compared to human COPD. Lymphocytes made up less than 2% of the total cells. Lastly, whether the damage induced in this model is reversible has not been tested.

In conclusion, tobacco smoke-induced lung inflammation is the cause of most COPD cases. These findings have important implications for understanding the etiology of COPD and suggest that pharmaceuticals designed to reduce leukocyte recruitment through the bronchial circulation may have promise as potential therapy to treat COPD.
